# Understanding the relationship of academic motivation and social support in graduate nursing education in Egypt

**DOI:** 10.1186/s12912-023-01671-5

**Published:** 2024-01-02

**Authors:** Mona Metwally El-Sayed, Samah Mohamed Taha, Eman Sameh AbdElhay, Manal Mohammed Hawash

**Affiliations:** 1 Psychiatric and Mental Health Nursing, Faculty of Nursing, Alexandria University, Alexandria, Egypt; 2 Psychiatric and Mental Health Nursing, Nursing Faculty, Mansoura University, Mansoura, Egypt; 3 Gerontological Nursing, Faculty of Nursing, Alexandria University, Alexandria, Egypt

**Keywords:** Academic motivation, Social Support, Graduates, Nursing education, Egypt

## Abstract

**Background:**

Obtaining a postgraduate nursing education in Egypt requires substantial resources, including a robust academic drive and supportive relationships. These resources help students overcome challenges and navigate their educational journey. This study examines the relationship between academic motivation and social support among nursing graduate students.

**Design:**

A cross-sectional survey was conducted at two nursing colleges in Egypt, involving 410 randomly selected graduate students.

**Measures:**

The Academic Motivation Scale—College Version (AMS-C 28) and the Multidimensional Scale of Perceived Social Support (MSPSS) were used.

**Findings:**

Findings revealed a statistically significant positive relationship between academic motivation and perceived social support (r = 0.515, *p* < 0.001). Hierarchical regression analysis revealed that 12.9% (Adjusted R² = 0.129) of the variance in academic motivation could be explained by perceived social support and level of education.

**Conclusion:**

This study pinpointed intrinsic motivation as the primary impetus for graduate nursing students. Robust social support and active participation in social and recreational activities were also significant motivators. Nursing education programs can enhance intrinsic motivation by integrating authentic learning experiences, providing empowering mentorship, offering condensed doctoral programs, and ensuring substantial peer support. The exploration of the role of novelty in graduate nursing education, facilitated by innovative teaching methods such as collaborative virtual reality simulations, gamification, and team-based research projects, can be advantageous. Building robust social networks is vital for establishing a supportive and motivating learning environment for nursing graduates in Egypt.

## Introduction

Graduate nurses face numerous hurdles during their academic journey, including demanding academic workloads, rapid advancements in medical technology, substantial responsibilities, and limited mentorship and financial resources. While some nurses navigate these challenges effectively, others struggle persistently, highlighting the critical need for adequate support from educational institutions and healthcare organizations [1, 2].

Academic motivation, as defined by Bozanoglu (2004), is the driving force behind academic engagement and activities [3]. Self-determination theory emphasizes its pivotal role in academic growth and performance, highlighting its ability to fuel energy, self-regulation, and overall learning capacity [4]. Academic motivation can be categorized into intrinsic and extrinsic types [4]. Intrinsic motivation originates from within, driven by the inherent enjoyment and satisfaction of learning and personal growth [3, 5]. On the other hand, extrinsic motivation is fueled by external rewards, such as grades, recognition, or avoidance of punishment [5, 6].

Both intrinsic and extrinsic motivation play critical roles in encouraging graduate nurses to pursue their studies [5, 7]. Intrinsic factors often include a strong desire for personal growth, achievement, and knowledge acquisition [8]. Self-perceived competence, as emphasized by the Self-Determination Theory, is crucial for shaping motivational behaviors and encouraging goal completion [4]. Graduate nurses who perceive themselves as capable and effective learners are more likely to invest significant effort in achieving their academic objectives [4]. Robust intrinsic motivation significantly reduces the likelihood of program dropout in graduate nursing education [9].

Extrinsic factors such as family support, professional aspirations, program duration, and the COVID-19 pandemic can influence academic motivation [7, 10–12]. The pandemic has emerged as a significant extrinsic factor impacting graduate nurses’ motivation in Egypt, leading to decreased control over learning, reduced engagement preferences, and diminished feelings of competence and autonomy, primarily due to the shift to online learning formats [13, 14].

Social support from family, teachers, friends, and peers significantly enhances academic motivation [15–17]. In Egypt, the social integration of graduate nursing candidates profoundly impacts their study motivation, mental well-being, and academic performance [15, 16]. Studies investigating the relationship between social integration, support, and academic motivation reveal a synergistic effect, significantly amplifying motivational levels [18, 19]. Social support acts as a protective factor, fostering feelings of security and contributing to overall physical and mental well-being [18]. Research also suggests that students’ motivational needs and perceived support may vary throughout their academic journey [3, 6, 20]. Notably, graduate nursing candidates often exhibit high motivation levels at the program’s offset, which tend to decrease as they progress [11, 12, 16].

Previous studies primarily focused on undergraduate nursing education, overlooking the unique dynamics [15, 16]. While existing research has independently explored academic motivation and perceived social support in various contexts, their interplay within the specific environment of graduate nursing education in Egypt still needs to be explored. This research gap necessitates a deeper understanding of how these factors interact and influence graduate nursing candidates’ experiences. This study addresses this critical need by investigating the relationship between academic motivation and PSS in Egypt’s graduate nursing education context. Uncovering valuable insights into how graduate nursing students perceive their motivation and access to support can inform the development of effective programs and interventions. These interventions can bolster academic motivation, enhance performance, and ultimately contribute to advancing graduate nursing education in Egypt. This study aimed to achieve this goal by exploring the relationship between academic motivation and PSS among graduate nursing candidates in Egypt.

### Research questions

- What are the levels of academic motivation and perceived social support among graduate nursing candidates?
- What is the relationship between academic motivation and perceived social support among graduate nursing candidates?
- Which factors predict academic motivation among graduate nursing candidates?

## Methods

### Study design and setting

A cross-sectional survey was conducted between September 30th and October 30th, 2023, at two nursing colleges affiliated with the Ministry of Higher Education in Egypt: El-Mansoura University (College A) and Alexandria University (College B). These colleges are affiliated with the Ministry of Higher Education and comprise nine scientific departments. They were chosen due to their pioneering role in the field of nursing education in Egypt and their ability to accommodate many graduate candidates. These colleges offer flexible and comprehensive graduate programs structured on a credit hour system, culminating in a nursing license after the bachelor’s degree and further specialization options through a one-year diploma, a three-year master’s, and a five-year doctoral program, progressively deepening expertise in the chosen field.

### Participants

#### Inclusion and exclusion criteria

The study included graduate candidates enrolled in the College of Nursing at El-Mansoura University (A) and Alexandria University (B) for the 2023–2024 academic years. The participants in the study were newly registered in graduate nursing programs. These programs included a diploma, master’s, or doctorate in nursing. Candidates who failed to pass the required courses were excluded from the study. In addition, voluntary participation in the research was a requirement for inclusion. Graduate candidates with pre-existing psychiatric disorders, including Major Depressive Disorder, Bipolar I or II Disorder, Generalized Anxiety Disorder, or any other, were excluded from the study, along with those receiving medication or undergoing therapy for such conditions.

#### Sample size estimation

According to the data obtained from the Office of Graduate Studies and Research at two nursing colleges, 1251 (A = 541 and B = 710) graduate candidates were enrolled for the current academic year. The sample size for the study was determined using the G*Power Windows 3.1.9.7 software, considering various measures: statistical power (1-β error probability) of 0.95, an effect size of 0.5, significance level (α-error probability) of 0.01, number of groups of 1, and number of predictors of 1. The software calculated a minimum sample size of 384 candidates necessary for the study. Considering a 10% anticipated unresponsive rate, the final sample size was 410 candidates.

#### Sampling method and recruitment

**Fig. 1:**
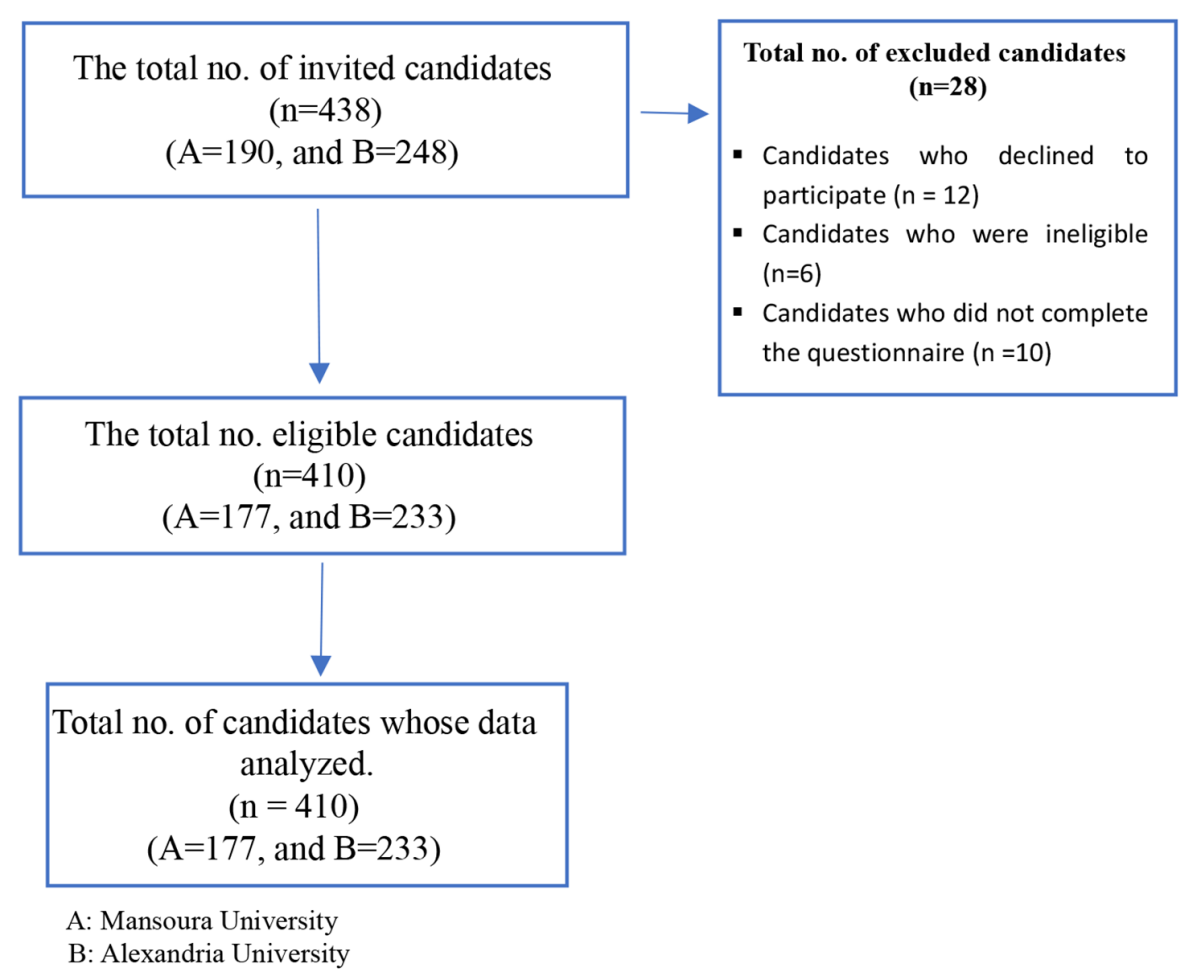
Recruitment flowchart

The research team began by acquiring enrollment data for two colleges, totaling 1251 graduate candidates (541 in College A and 710 in College B), after obtaining permission to access the registration lists from both colleges’ Candidates’ Affairs Department. To ensure proportional representation from each college, the researchers employed stratified sampling, randomly selecting 190 candidates from College A and 248 from College B using Random Generator software. Following the selection, 438 candidates were invited to participate in the study. While 12 declined and 6 were deemed ineligible, 10 additional candidates did not complete questionnaires and withdrew, resulting in a final sample size of 410 (177 from College A and 233 from College B). These final participants formed the basis for the subsequent analysis (Fig. 1).

### Measures

#### Graduate candidates’ sociodemographic data sheet

The researchers created a questionnaire to elicit information on the sociodemographic characteristics of the participants in the study, including their age, gender, place of residence, living status, education levels, and social and recreational activities.

#### Arabic version of academic motivation scale (AMS-C 28) college version

The AMS-C28 self-report questionnaire measures academic motivation [6]. The questionnaire comprises 28 items, which are organized into 7 sub-scales. These sub-scales consist of three components related to intrinsic motivation: (1) intrinsic motivation—to know; (2) intrinsic motivation—towards accomplishment; and (3) intrinsic motivation—to experience stimulation. Additionally, three sub-scales focus on extrinsic motivation: (4) extrinsic motivation—identified; (5) intrinsic motivation—introjected; (6) extrinsic motivation—external regulation. Lastly, the seventh sub-scale addresses the motivation aspect of the questionnaire. Participants rate their agreement with each statement on a 7-point Likert-type scale, extending from 1 (not at all true) to 7 (very true). To calculate the total score, the scores from each subscale are summed so they can be varied from 28 to 196. The standardized Arabic version [21] of the scale was utilized and demonstrated reasonable internal consistency (α = 0.88).

#### Arabic version of the multidimensional scale of perceived social support (MSPSS)

The MSPSS is a self-report questionnaire designed to measure an individual’s perceptions of social support adequacy from family, friends, and a significant other [22]. It contains 12 items rated on a 7-point Likert scale from “very strongly disagree” to “very strongly agree.” Participants indicate the extent to which they agree with statements related to social support, such as “My family tries to help me” or “I can talk about my problems with my friends.” The MSPSS has three subscales, with four items each, assessing perceived support from family, friends, and a significant other. Mean scores on the subscales and total scale are calculated by summing items and dividing by 4 or 12, respectively, and the total score varies from 1 to 7. The standardized Arabic version [22, 23] showed an excellent Cronbach’s alpha reliability of 0.91.

### Procedures and data collection

#### Ethical agreement

The required official authorization to conduct the study was obtained from the Research Ethical Committee (REC) of the College of Nursing at El-Mansoura University. Endorsements were obtained from the respective authorities of the two nursing colleges for gathering data. After clarifying the purpose of the study, participants were informed and provided with written informed consent. The questionnaire was anonymous, and the data were treated with confidentiality. Each participant was provided with information regarding their freedom to withdraw their participation from the study at any time without any negative consequences.

#### A pilot study and reliability

After obtaining the necessary approvals from the relevant authorities, a pilot survey was conducted involving 40 participants who met the established eligibility criteria. Of these, 20 participants were selected from Alexandria University and another 20 from El Mansoura University. The primary objective of the pilot study was to assess the objectivity, transparency, feasibility, and applicability of the research instruments. The questionnaires were suitable and implemented based on the pilot study’s findings. It is important to note that the participants who participated in the pilot survey were excluded from the final study sample. The reliability of the instruments was tested through a Cronbach’s alpha coefficient test, employed at a significance level of *p* ≤ 0.05.

### Data collection

After excluding participants from the pilot study, a representative sample was recruited using a simple random sampling technique. Trained researchers then conducted structured interviews with each participant and collected the required data using the approved data collection instruments. Each interview was conducted in an empty classroom, where one student was interviewed for approximately 20–25 min to ensure privacy. Informed consent was obtained, guaranteeing anonymity and confidentiality. The interviewer had no authority over the students, and participation was not incentivized. The graduates were assured of voluntary participation and the right to withdraw without penalty. Their responses were kept confidential. To ensure the completeness of the information, the researchers thoroughly reviewed the participant’s responses to the data collection tool.

### Data processing

The data were coded and entered into IBM SPSS (Statistical Package for the Social Sciences) version 26. Following data entry, review, and verification, the process was repeated to ensure no errors occurred during data input. Kolmogorov-Smirnov tests were used to assess the normality of the quantitative variable distribution. Cronbach’s alpha coefficients were used to assess the internal consistency of two quantitative variables. The participants’ academic motivation and perceived social support were presented using means (M) and standard deviations (SD), as well as frequencies and percentages. The correlation between these variables was assessed using the Pearson correlation coefficient. A hierarchical regression analysis was conducted to measure the variance in the level of academic motivation based on the perception of social support and other demographic factors. All statistical tests were conducted at a significant level of 0.05.

## Results

Table 1 displays the sociodemographic characteristics, academic motivation, and participants’ perceived social support. Most of the participants (71.0%) were female. In terms of age, 67.1% of them were 25 years or older. Most participants (84.0%) resided in urban areas, and 97.0% lived with their families. Regarding educational levels, 32.4% were enrolled in a nursing diploma program, 45.9% in a master’s program, and 21.7% were pursuing a doctorate. Regarding social and recreational activities, a significant majority (94.6%) of the participants engaged in such activities. The most popular activities included visiting relatives and friends (78.3%), going to social clubs (69.8%), and going on trips (63.4%). Additionally, 29.8% of the participants reported engaging in sports activities, and 31.2% participated in art and music activities. The average academic motivation and perceived social support scores were 88.54 (SD = 30.07) and 5.20 (SD = 1.30).

**Table 1:** Sociodemographic characteristics, academic motivation, and perceived social support among the participants

Variables	Categories	Total (n=410)	
		***N***	***%***
Sex	Male	119	29.0
	Female	291	71.0
Age	<25	135	32.9
	25 –	275	67.1
Residence	Urban	336	84.0
	Rural	74	16.0
Living Status	With family	398	97.0
	Alone	12	3.0
Educational levels	Nursing Diploma	133	32.4
	Nursing Master	188	45.9
	Nursing Doctorate	89	21.7
# Social and Recreational Activities	Yes	388	94.6
	No	22	5.4
	Visiting relatives and friends	321	78.3
	Going to social clubs	286	69.8
	Doing sports	122	29.8
	Doing art and music activities	128	31.2
	Going to trips	260	63.4
Academic Motivation	Mean (SD)	88.54	30.07
Perceived Social Support	Mean (SD)	5.20	1.30

# multiple responses

Table 2 delves into the relationships between various dimensions of academic motivation and perceived social support (PSS), addressing the second research question. The findings reveal significant positive correlations between intrinsic motivation (to know, accomplish, and experience stimulation) and PSS (r = 0.554, 0.265, and 0.472, respectively; *p* < 0.001 for all). Turning to extrinsic motivation, identified regulation, introduced regulation, and external regulation demonstrate statistically significant positive correlations with PSS (r = 0.423, 0.472, and 0.280, respectively; *p* < 0.001 for all). Conversely, motivation exhibits a statistically significant negative correlation with PSS (r = -0.199, *p* < 0.001).

**Table 2:** Relationship between different dimensions of motivation and perceived social support

AMS-C 28		Intrinsic motivation – to Know	Intrinsic motivation – towards accomplishment	Intrinsic motivation –to experience stimulation	Extrinsic motivation- identified	Extrinsic motivation— introjected	Extrinsic motivation- external regulation	A motivation	Total of AMS-C28	Family Support	Friends Support	Significant Others Support	Total of MSPSS
**Intrinsic** **motivation –** **to Know**	**r** **P**		0.288** < 0.001	0.348** < 0.001	0.369** < 0.001	0.344** < 0.001	0.378** < 0.001	0.368** < 0.001	0.228** < 0.001	0.146** 0.003	0.206** < 0.001	0.164** < 0.001	0.554** < 0.001
**Intrinsic** **motivation –** **towards accomplishment**	**r** **P**			0.415** < 0.001	0.258** < 0.001	0.634** < 0.001	0.428** < 0.001	0.412** < 0.001	0.413** < 0.001	0.160** 0.001	0.148** 0.003	0.147** 0.003	0.265** < 0.001
**Intrinsic** **motivation –** **to experience stimulation**	**r** **P**				0.428** < 0.001	0.561** < 0.001	0.391** < 0.001	0.368** < 0.001	0.508** < 0.001	0.255** < 0.001	0.338** < 0.001	0.268** < 0.001	0.472** < 0.001
**Extrinsic** **motivation-** **identified**	**r** **p**					0.367** < 0.001	0.421** < 0.001	0.452** < 0.001	0.621** < 0.001	0.238** < 0.001	0.354** < 0.001	0.269** < 0.001	0.423** < 0.001
**Extrinsic motivation—** **introjected**	**r** **p**						0.578** < 0.001	0.528** < 0.001	0.369** < 0.001	0.212** < 0.001	0.339** < 0.001	0.222** < 0.001	0.472** < 0.001
**Extrinsic** **motivation-** **external** **regulation**	**r** **P**							0.469** < 0.001	0.258** < 0.001	0.428** < 0.001	0.516** < 0.001	0.469** < 0.001	0.280** < 0.001
**A motivation**	**r** **P**								0.459** < 0.001	0.214** < 0.001	0.292** < 0.001	0.204** < 0.001	− 0.199** < 0.001
**Total of** **AMS-C 28**	**r** **P**									0.279** < 0.001	0.398** < 0.001	0.303** < 0.001	0.515** < 0.001
**Family Support**	**r** **p**										0.816** < 0.001	0.856** < 0.001	0.949** < 0.001
**Friends Support**	**r** **p**											0.814** < 0.001	0.937** < 0.001
**Significant Others** **Support**	**r** **p**												0.935** < 0.001
**Total of MSPSS**	**r** **p**												

AMS-C 28: Academic Motivation Scale-College Version

MSPSS: Multidimensional Scale of Perceived Social Support

r: the Pearson correlation coefficient

* Correlation is significant at the 0.05 level (2-tailed). ** Correlation is significant at the 0.01 level (2-tailed)

Finally, the overall score of academic motivation displays statistically significant positive relationships with the family, friends, and significant other support subscales, and the total PSS score (r = 0.279, 0.398, 0.303, and 0.515, respectively; *p* < 0.001 for all).

Table 3 explores the influence of demographic characteristics on academic motivation. Sex, age, residence, living status, and general participation in social and recreational activities do not significantly impact academic motivation. However, a statistically significant score difference emerges based on educational level (F = 4.014, *p* = 0.019). Specifically, candidates holding a nursing diploma exhibited the highest average score. Additionally, within the category of social and recreational activities, the type of activity engaged in demonstrates a significant difference in scores (F = 4.860, *p* = 0.001). Sports, art, and music activities had the highest average scores.

**Table 3:** Relationship between sociodemographic characteristics of the participants and academic motivation (n = 410)

Variables	Categories	Academic motivation	Test of sig.
		M (SD)	
Sex	Male	86.71 (19.59)	t = 1.293 *p* = 0.197
	Female	89.36 (18.47)	
Age	< 25	86.63 (19.10)	t = 1.500 *p* = 0.134
	25 –	89.58 (18.63)	
Residence	Urban	88.05 (19.36)	t = 1.178 *p* = 0.239
	Rural	90.85 (16.35)	
Living Status	With family	88.66 (18.64)	t = 0.361 *p* = 0.718
	Alone	86.67 (24.84)	
Educational levels	Nursing Diploma	92.33 (15.47)	F = 4.014* *p* = 0.019
	Nursing Master	87.0 (20.57)	
	Nursing Doctorate	86.66 (19.83)	
Social and Recreational Activities	Yes	88.6119.15	t = 0.032 *p* = 0.975
	No	88.48 (12.04)	
	Visiting relatives and friends	88.58 (19.28)	F = 4.860* *p* = 0.001
	Going to social clubs	88.93 (18.16)	
	Doing sports	93.62 (15.32)	
	Doing art and music activities	93.24(15.38)	
	Going to trips	86.29 (19.91)	

M: Mean SD (Standard Deviation)

F: One-way ANOVA test t: Student t-test *: Statistically significant at *p* ≤ 0.05

Table 4 presents findings from the hierarchical regression analysis, examining predictors of academic motivation. In the first step, perceived social support emerges as a significant positive predictor, explaining approximately 12.2% of the variance in academic motivation (Adjusted R² = 0.120). The model’s constant term is 61.082, with a statistically significant t-value of 16.269 (*p* < 0.001). The unstandardized coefficient (B) is 5.591, while the standardized coefficient (Beta) is 0.350, indicating a 5.591 increase in academic motivation for every unit increase in perceived social support. The second step incorporates both perceived social support and educational levels as predictors. This statistically significant model accounts for approximately 12.9% of the variance in academic motivation (Adjusted R² = 0.129). The constant term is 66.461, with a statistically significant t-value of 15.098 (*p* < 0.001). Notably, educational level emerges as a significant negative predictor in the model. Its unstandardized coefficient (B) is -2.748, its standardized coefficient (Beta) is -0.107, and its p-value is 0.021. This indicates that holding all other variables constant, academic motivation decreases as the level of education increases. This finding suggests that doctoral candidates experienced lower levels of academic motivation.

**Table 4:** A hierarchical regression analysis between academic motivation, perceived social support, and other covariates. (n = 410)

	Model	Unstandardized Coefficients		Standardized Coefficients	t	Sig.	F	Sig.	R	R^2^	Adjusted R^2^
		**B**	**Std. Error**	**Beta**							
Step1	**(Constant)**	61.082	3.755		16.269	< 0.001*					
	**MSPSS**	5.591	0.742	0.350	7.535	< 0.001*	56.777 *	< 0.001*	0.350	0.122	0.120
Step2	**(Constant)**	66.461	4.402		15.098	< 0.001*					
	**MSPSS**	5.551	0.738	0.347	7.519	< 0.001*	31.354	< 0.001*	0.365	0.134	0.129
	**Educational levels**	-2.748	1.191	-0.107	-2.308	0.021*					

MSPSS: Multidimensional Scale of Perceived Social Support

a.: dependent variable (Academic Motivation); b: independent variable (Perceived Social Support, Educational levels)

F (ANOVA)

R2: Coefficient of determination

B: Unstandardized Coefficients

Beta: Standardized Coefficients

t: t-test of significance

LL: Lower limit UL: Upper Limit

*: Statistically significant value at *p* ≤ 0.01

## Discussion

Graduate nursing education demands an equally strong, if not more robust, academic motivation than other disciplines. Delivering high-quality nursing services necessitates equipping nursing candidates with a persistent desire to learn and adapt as the field advances, alongside the drive to acquire extensive knowledge and skills [24]. Motivation is crucial in directing individuals toward their goals, and its strength or weakness significantly impacts goal achievement [25]. Identifying the factors influencing academic motivation across diverse academic groups and levels can pave the way for strategies to eliminate, mitigate, or enhance these motivating aspects. Therefore, this study explored the relationship between academic motivation and perceived social support among graduate nursing candidates.

Graduate candidates in the study generally displayed moderate levels of academic motivation. Their scores were high in “intrinsic motivation towards accomplishment” and “intrinsic motivation to know,” indicating a strong desire for personal achievement and intellectual growth. However, their interest in seeking novelty or excitement was slightly lower. Their scores on extrinsic motivation (identified, introduced, and external regulation) were also moderate. This could be attributed to the specific nature of graduate studies, which often require high levels of self-direction and intrinsic motivation to succeed. The structured nature of nursing academic programs also limits opportunities for novel experiences that might otherwise enhance extrinsic motivation. Additionally, the rewards of graduate studies, such as career advancement or increased earning potential, are often delayed, potentially diminishing their motivational impact.

Moreover, the majority of the participants being female could influence these results, as previous research suggests possible gender differences in motivation. This aligns with the findings by Adib et al. (2019), who identified a strong positive correlation between academic motivation and self-directed learning [26]. Since self-directed learners are known for their strong desire to learn, motivation becomes crucial to their success. Vahedian-Azimi and Moayed (2021) conducted a cross-sectional study involving 220 graduate nursing candidates across ten nursing schools. Using the Vallerand Academic Motivation Scale, they investigated the relationship between academic motivation and self-esteem. Their findings indicated that graduate nursing candidates demonstrated moderate motivation to study, with 57.3% achieving scores exceeding 129 out of 196 on the academic motivation scale [27].

Conversely, Fatima et al. (2021) found that nursing candidates exhibited higher levels of extrinsic than intrinsic motivation [28]. The primary drivers of their extrinsic motivation were the desire for a well-paying career and a comfortable life. Elbsuony (2016) also conducted a study in Saudi Arabia that revealed poor academic motivation among nurses [29].

This study found that, although no significant differences were observed in academic motivation based on gender, age, residence, or living status, educational level emerged as a key influencer. Notably, candidates enrolled in nursing diploma programs demonstrated higher average academic motivation than those in master’s and doctorate programs. This finding was further supported by the observation that educational level emerged as a significant negative predictor, accounting for 12.9% of the variance in academic motivation (adjusted R² = 0.129). This means that academic motivation tends to decrease as the level of education increases.

Several potential explanations exist for this phenomenon. Firstly, transitioning from the practical, hands-on curriculum of nursing diploma programs to the more research-oriented environment of master’s and doctorate programs can be challenging for many candidates. These higher-level programs often require a strong foundation in research methodology, which may not be emphasized in nursing diploma programs. This can lead to a steep learning curve for candidates, potentially impacting their motivation. Secondly, the emphasis on independent study and self-directed learning in higher-level programs can be problematic for students accustomed to structured learning environments. This shift in learning style can require significant adaptation and adjustment, which may present challenges for some candidates.

Finally, the pressure to produce original research and contribute to the academic community can be daunting for many candidates, particularly those in master’s and doctorate programs. This pressure can negatively impact their motivation and overall academic performance.

These findings highlight the importance of understanding the factors influencing academic motivation among graduate nursing candidates. By recognizing the specific challenges candidates face in different educational programs, we can develop interventions and tailored teaching approaches to better support their needs and foster a more positive learning environment. Additionally, investigating the impact of research methodology training and exploring strategies to assist candidates in transitioning to independent learning styles hold promise for further enhancing academic motivation in this population.

These findings aligned with Gumusgul and Gumugul (2019), who demonstrated that academic motivation and gender were not statistically significant [30]. Age and the motivation sub-dimension showed significant differences. It has been noted that motivation decreases with aging. It is believed that while older candidates understand the long-term benefits of education, younger candidates are more motivated to excel academically. Conversely, according to Heidarian et al. (2015), years of service, gender, age, hiring status, marital status, and academic degree are significant variables that affect motivating factors [31]. Furthermore, recreational sports participation and academic motivation reveal significant differences between the participants. Engaging in physical and recreational activities is a powerful extrinsic motivator that fosters a sense of autonomy and self-direction and may inspire future planning.

The present study’s findings reveal that most graduate nursing candidates reported experiencing moderate levels of perceived social support (PSS) across the three categories of family, friends, and significant others. Despite residing in urban areas with their families and maintaining regular contact with relatives, participants’ perceptions of support remained moderate. This may be attributed to the demanding nature of the nursing profession and the rigorous academic requirements of graduate-level studies. Additionally, the availability and accessibility of academic support and guidance at the university may influence perceived social support levels. These factors can shape how individuals perceive the support they receive, ultimately resulting in the observed moderate levels of PSS. These findings align with those of Abdel El-Halem (2011) [16] and Jeong and Koh (2021) [32], who emphasized the crucial role of family, friends, colleagues, and academic support in the lives of graduate nursing candidates.

Conversely, when individuals lack expected support from their network, feelings of isolation during graduate studies may intensify. This underscores the critical need for sufficient support for graduate nursing candidates. In contrast, Radeef and Faisal (2020) found that undergraduate nursing students reported experiencing higher levels of social support [33].

This study revealed a positive association between academic motivation and support from friends, family, and significant others (r = 0.279, 0.398, 0.303, *p* < 0.001). Furthermore, our stepwise regression analysis demonstrates that perceived social support (PSS) significantly predicts academic motivation, accounting for 12.2% of the variance (adjusted R² = 0.120). This association could be attributed to the diverse roles of PSS, which encompass nurturing, empathy, encouragement, information provision, material assistance, and sharing of experiences. Additionally, PSS can contribute to higher mastery goals, reduced performance-avoidance goals, decreased test anxiety, and enhanced academic motivation and achievement [32]. Social support, rather than its actual presence, can serve as a protective factor for individuals navigating through different stress levels, in line with the perception findings of Waleed & Ihab (2021) [34]. Their multiple regression analysis identified perceived social and family support (13.5%), social anxiety (1.9%), and self-esteem (1.1%) as the most significant factors explaining the variance in learning motivation. Similarly, Emadpoor ​​et al. (2016) found that academic motivation mediates and indirectly affects psychological well-being, accounting for 13% of the variance in academic motivation through PSS [35]. Moreover, Mostafa and Lim (2020) stated that social expectations, including the expectation of social support, increased social mobility, and positive student-teacher relationships, significantly influence female candidates’ academic motivation to pursue their degrees [36].

Despite the critical role of academic motivation and social support in nursing education, more research is needed to understand the relationship between these variables in the North Africa and the Middle East (MENA) region, especially among graduate nursing candidates. This study aims to bridge this gap by exploring these connections. Interestingly, a comprehensive review revealed only a handful of studies in Egypt that have produced similar findings, highlighting this research’s unique and significant contribution.

### Study limitations

The present study has some limitations that the researchers would like to acknowledge. First, our analysis relied on self-reported data regarding perceived social support and academic motivation. As such, biases in the measurement process may have impacted our findings. Additionally, the study was conducted with a relatively small sample size, potentially limiting the generalizability of the results. Future research employing longitudinal designs to track motivation over time and mixed-methods approaches to gain a more comprehensive understanding of academic motivation is recommended. Furthermore, investigations into other potential influencing factors, such as critical thinking, humor, teaching strategies, and academic and psychological counseling, are warranted to provide a more nuanced understanding of academic motivation and perceived social support.

### Conclusion and recommendations

This study revealed that intrinsic motivation, especially the desire to learn and achieve personal goals, was the primary driver for participants, while extrinsic motivation played a moderate role. Notably, academic motivation decreased with higher levels of education, requiring specific interventions for doctoral candidates. Additionally, engagement in social and recreational activities and perceived social support were significant positive predictors of motivation. Therefore, the study recommends interventions to enhance intrinsic motivation, address the decline among doctoral candidates, investigate the role of novelty and stimulation, integrate social and recreational activities, and strengthen perceived social support for graduate nursing candidates.

### Nursing implications

This study enriches our understanding of how their perceptions of social support shape graduates’ academic motivation. It can guide academics and researchers in exploring ways to boost graduate students’ motivation to learn. Developing and maintaining new, emotionally invested, and reliable support networks and strengthening pre-existing social bonds that encourage recognition of students’ abilities, competence, and value could be integral components of intervention programs to enhance academic motivation. In nursing education, programs can bolster intrinsic motivation by integrating authentic learning experiences, providing empowering mentorship, offering shorter doctoral programs, and ensuring strong peer support. Exploring novelty’s role in engagement, using innovative teaching methods such as collaborative virtual reality simulations, gamification, and team-based research projects can also be beneficial and foster a more enriching educational experience for graduates.

## Data Availability

The datasets used or analyzed in this study are available from the corresponding author upon request.

## Abbreviations

AM: Academic Motivation
SDT: Self-Determination Theory
PSS: Perceived Social Support
AMS-C: Academic Motivation Scale—College Version
MSPSS: Multidimensional Scale of Perceived Social Support
SPSS: Statistical Package for the Social Sciences
REC: Research Ethics Committee
